# Supplementary material for: Loss of the yeast transporter Agp2 upregulates the pleiotropic drug-resistant pump Pdr5 and confers resistance to the protein synthesis inhibitor cycloheximide
Source: PLoS One. 2024 May 22;19(5):e0303747. doi: 10.1371/journal.pone.0303747 (PMC11111045; doi:10.1371/journal.pone.0303747)
Supplement: S1 Fig — (PDF) [file pone.0303747.s001.pdf]

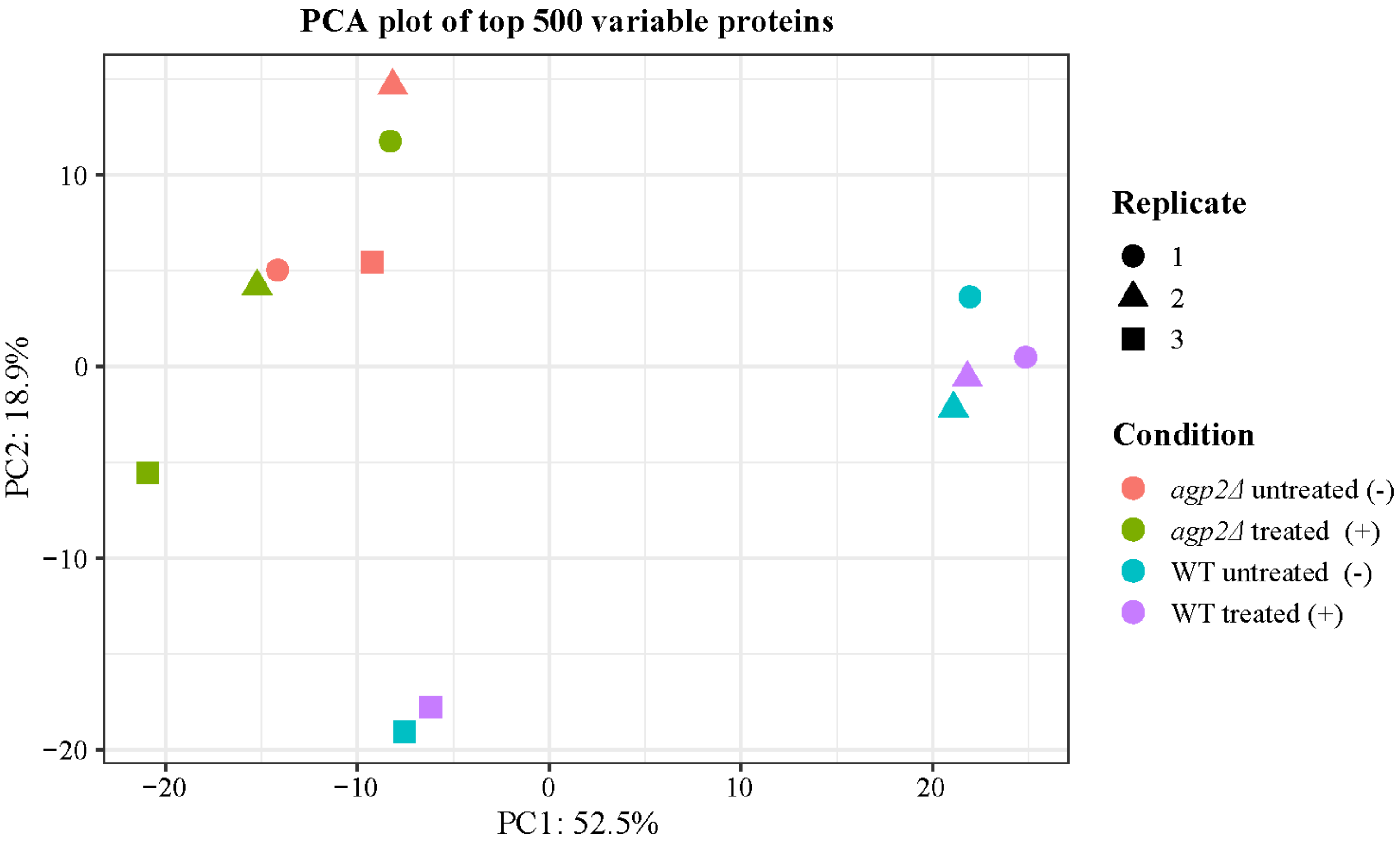

Supplementary Figure S1: PCA plot. Top 500 significantly variable protein across all the samples were used to generate a PCA plot. Each sample condition is represented with a different color as described in the legend. Each replicate is represented as a different shape as described in the legend.
