# Supplementary material for: Loss of the yeast transporter Agp2 upregulates the pleiotropic drug-resistant pump Pdr5 and confers resistance to the protein synthesis inhibitor cycloheximide
Source: PLoS One. 2024 May 22;19(5):e0303747. doi: 10.1371/journal.pone.0303747 (PMC11111045; doi:10.1371/journal.pone.0303747)
Supplement: S2 Fig — (PDF) [file pone.0303747.s002.pdf]

Cluster 1

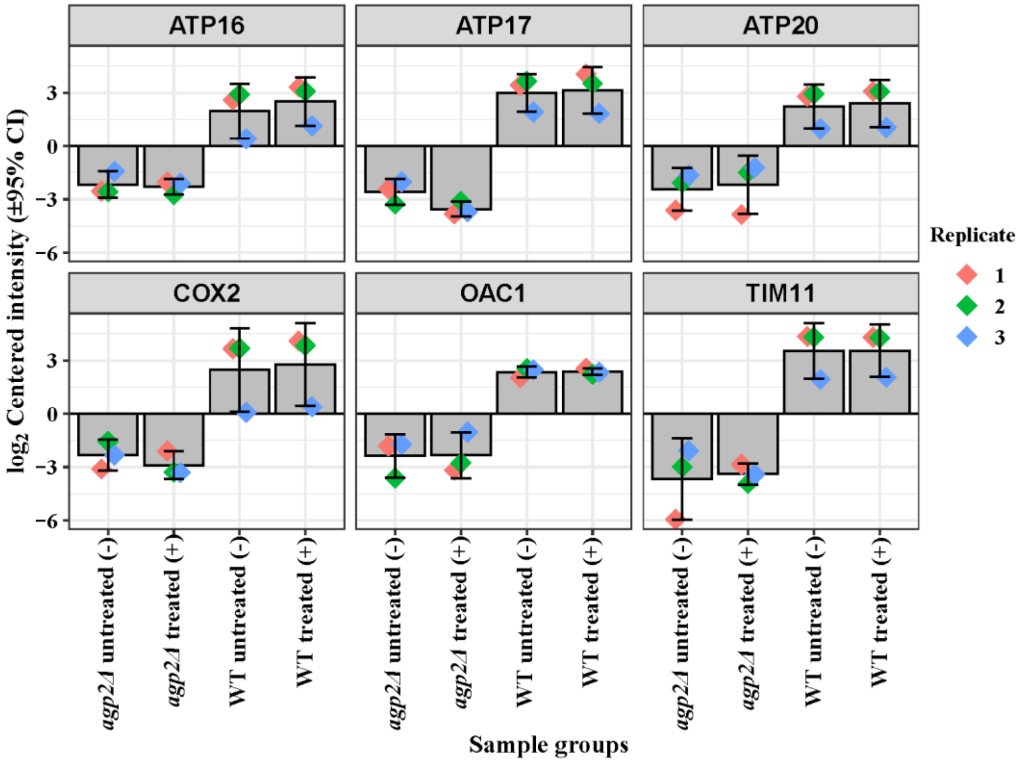
ATP16
ATP17
ATP20
COX2
OAC1
TIM11
log₂ Centered intensity (±95% CI)
3
0
−3
−6
agp2Δ untreated (-)
agp2Δ treated (+)
WT untreated (-)
WT treated (+)
Sample groups
Replicate
1
2
3

Cluster 2

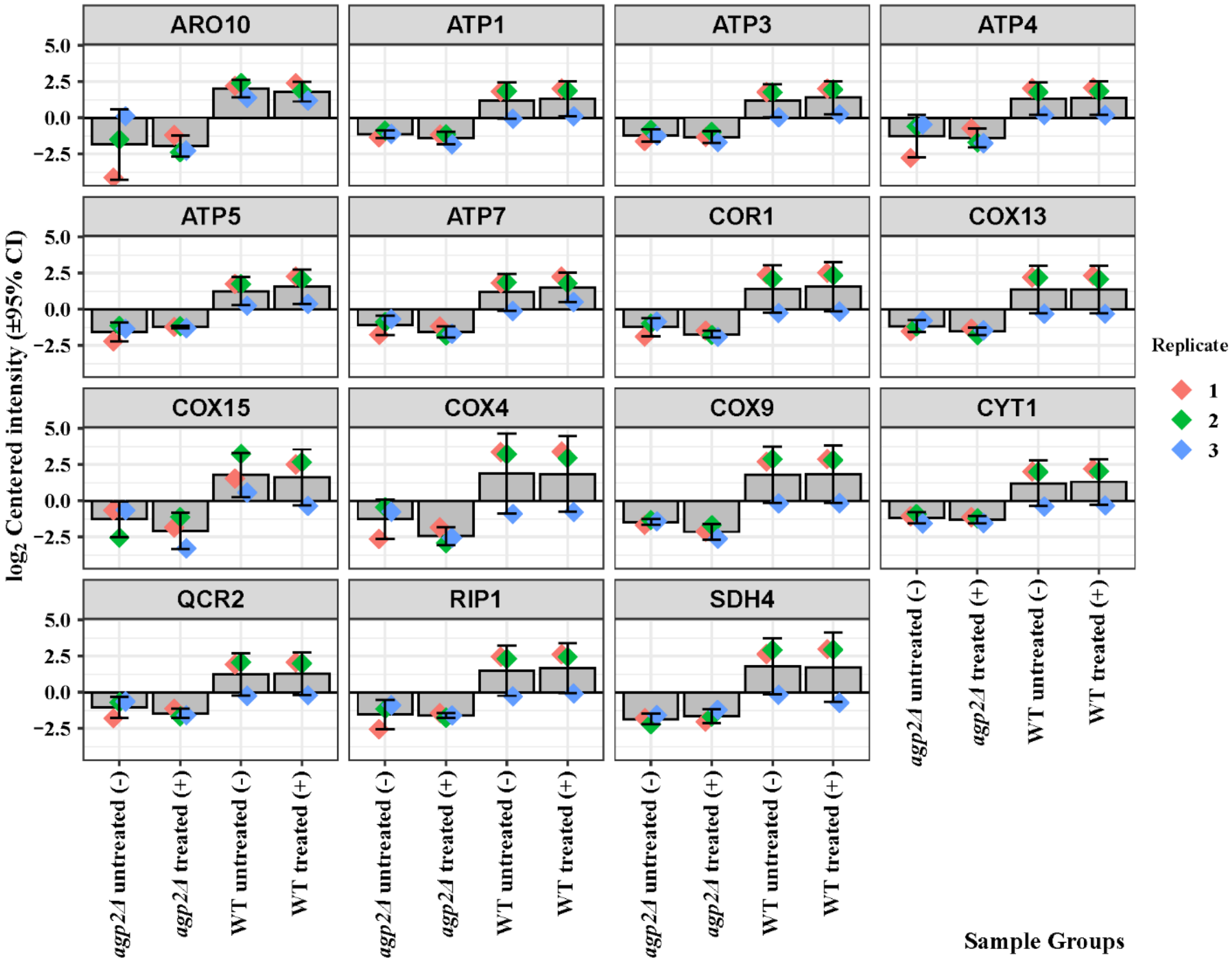
ARO10
ATP1
ATP3
ATP4
ATP5
ATP7
COR1
COX13
COX15
COX4
COX9
CYT1
QCR2
RIP1
SDH4
5.0
2.5
0.0
−2.5
log$_2$ Centered intensity (±95% CI)
Replicate
1
2
3
agp2Δ untreated (-)
agp2Δ treated (+)
WT untreated (-)
WT treated (+)
Sample Groups

Cluster 3

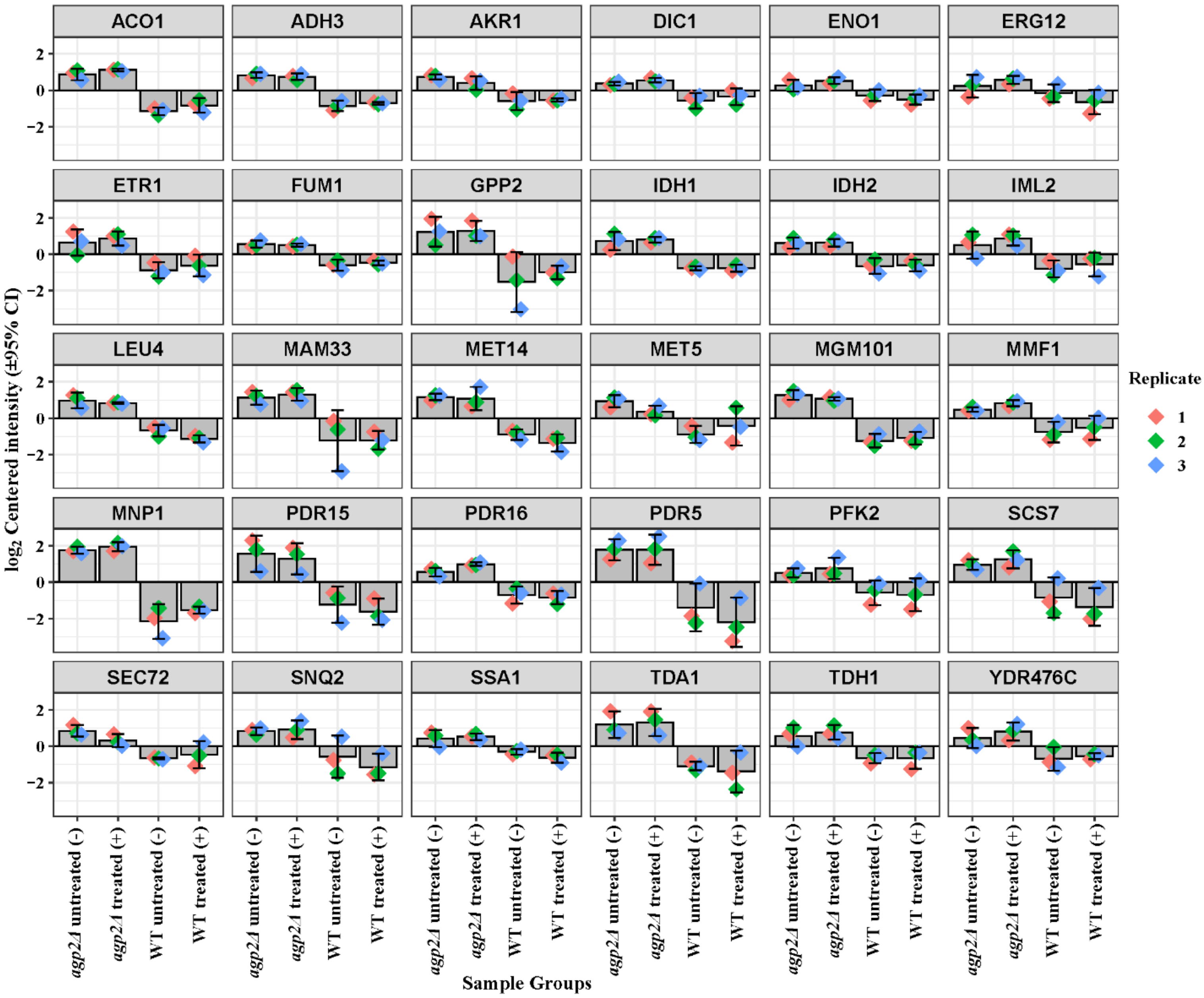
ACO1
ADH3
AKR1
DIC1
ENO1
ERG12
ETR1
FUM1
GPP2
IDH1
IDH2
IML2
LEU4
MAM33
MET14
MET5
MGM101
MMF1
MNP1
PDR15
PDR16
PDR5
PFK2
SCS7
SEC72
SNQ2
SSA1
TDA1
TDH1
YDR476C
2
0
−2
log_2 Centered intensity (±95% CI)
Replicate
1
2
3
agp2Δ untreated (-)
agp2Δ treated (+)
WT untreated (-)
WT treated (+)
Sample Groups

Cluster 4

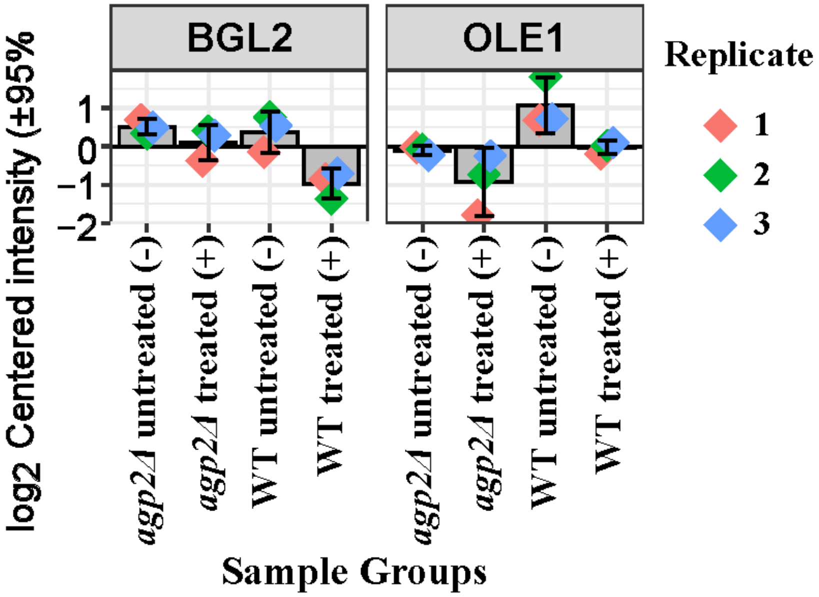
BGL2
OLE1
Replicate
1
2
3
log2 Centered intensity (±95%
1
0
−1
−2
agp2Δ untreated (-)
agp2Δ treated (+)
WT untreated (-)
WT treated (+)
agp2Δ untreated (-)
agp2Δ treated (+)
WT untreated (-)
WT treated (+)
Sample Groups

Cluster 5

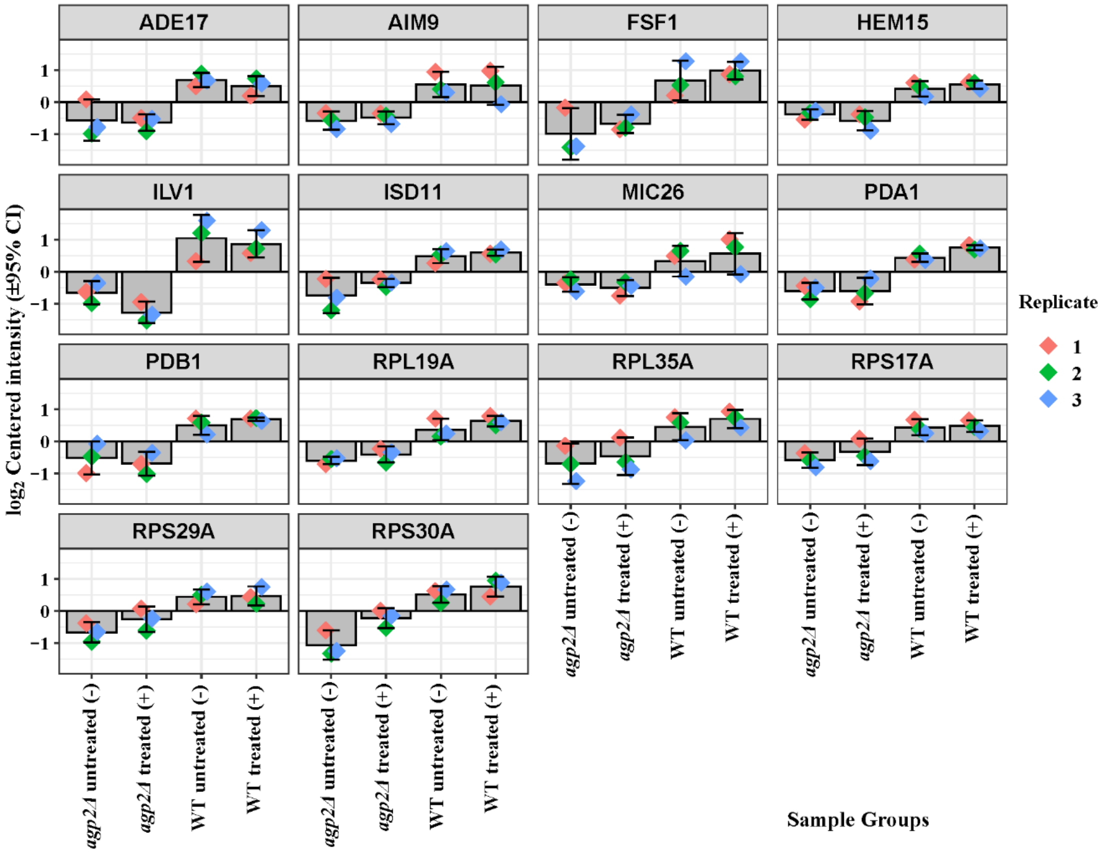
ADE17
AIM9
FSF1
HEM15
ILV1
ISD11
MIC26
PDA1
PDB1
RPL19A
RPL35A
RPS17A
RPS29A
RPS30A
log2 Centered intensity (±95% CI)
1
0
−1
agp2Δ untreated (-)
agp2Δ treated (+)
WT untreated (-)
WT treated (+)
Sample Groups
Replicate
1
2
3

Cluster 6

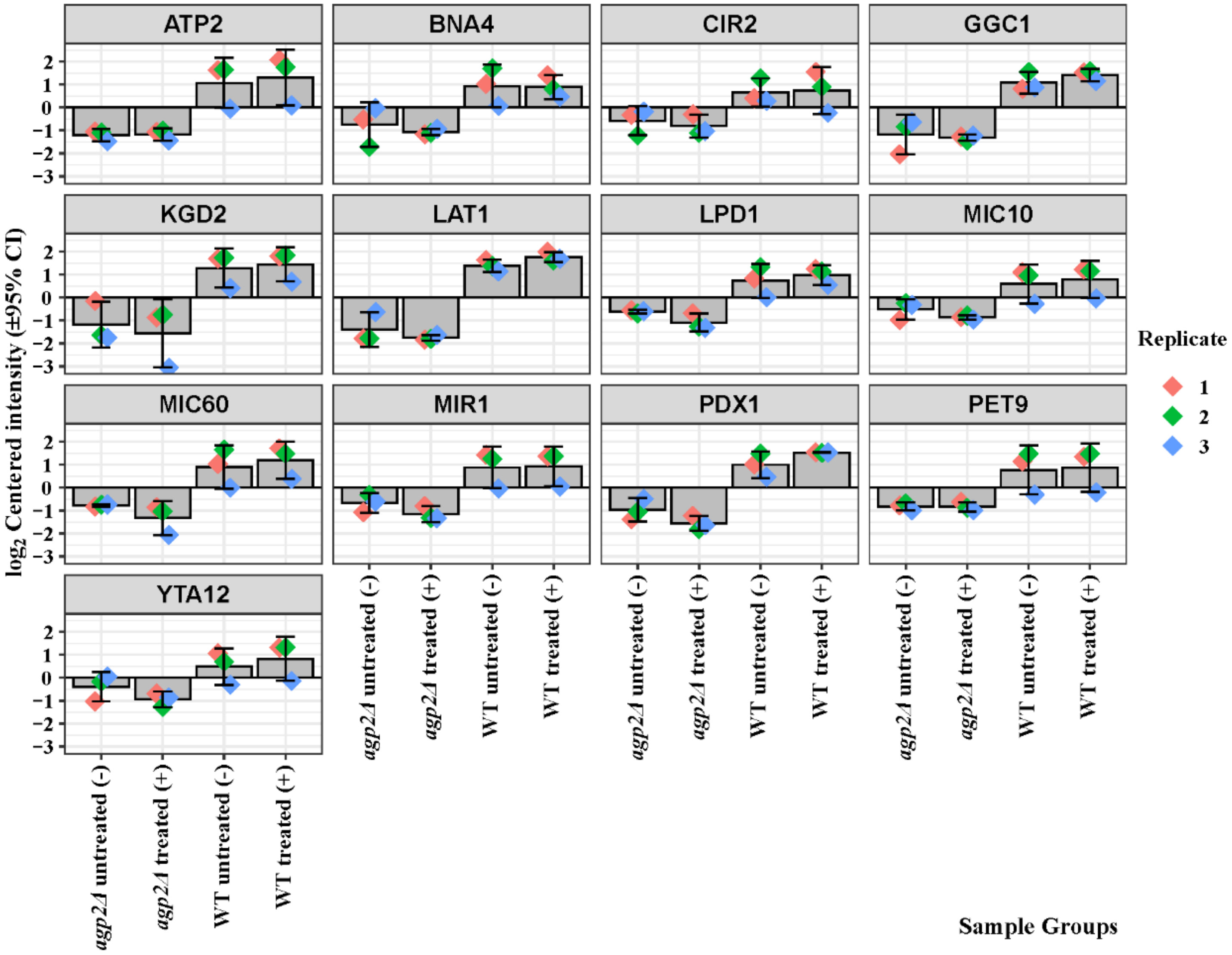

Supplementary Figure S2. Clusters 1 to 6 show the bar plots of the expressed proteins. Bar plots of Cluster 1, Cluster 2, Cluster 3 and Cluster 4 are protein repertoires with decreased expression in *agp2Δ* mutant. Bar plot of Cluster 5 showing the protein repertoire with increased expression in *agp2Δ* mutant. Cluster 6 represents proteins with mixed expression patterns in WT and the *agp2Δ* mutant sample groups. Each bar in the plot represents the data-centred log2 intensity of a protein across all the sample groups. Diamonds represent the log2 intensity value of each replicate with respect to the error bar.
