# Supplementary material for: Loss of the yeast transporter Agp2 upregulates the pleiotropic drug-resistant pump Pdr5 and confers resistance to the protein synthesis inhibitor cycloheximide
Source: PLoS One. 2024 May 22;19(5):e0303747. doi: 10.1371/journal.pone.0303747 (PMC11111045; doi:10.1371/journal.pone.0303747)
Supplement: S3 Fig — (PDF) [file pone.0303747.s003.pdf]

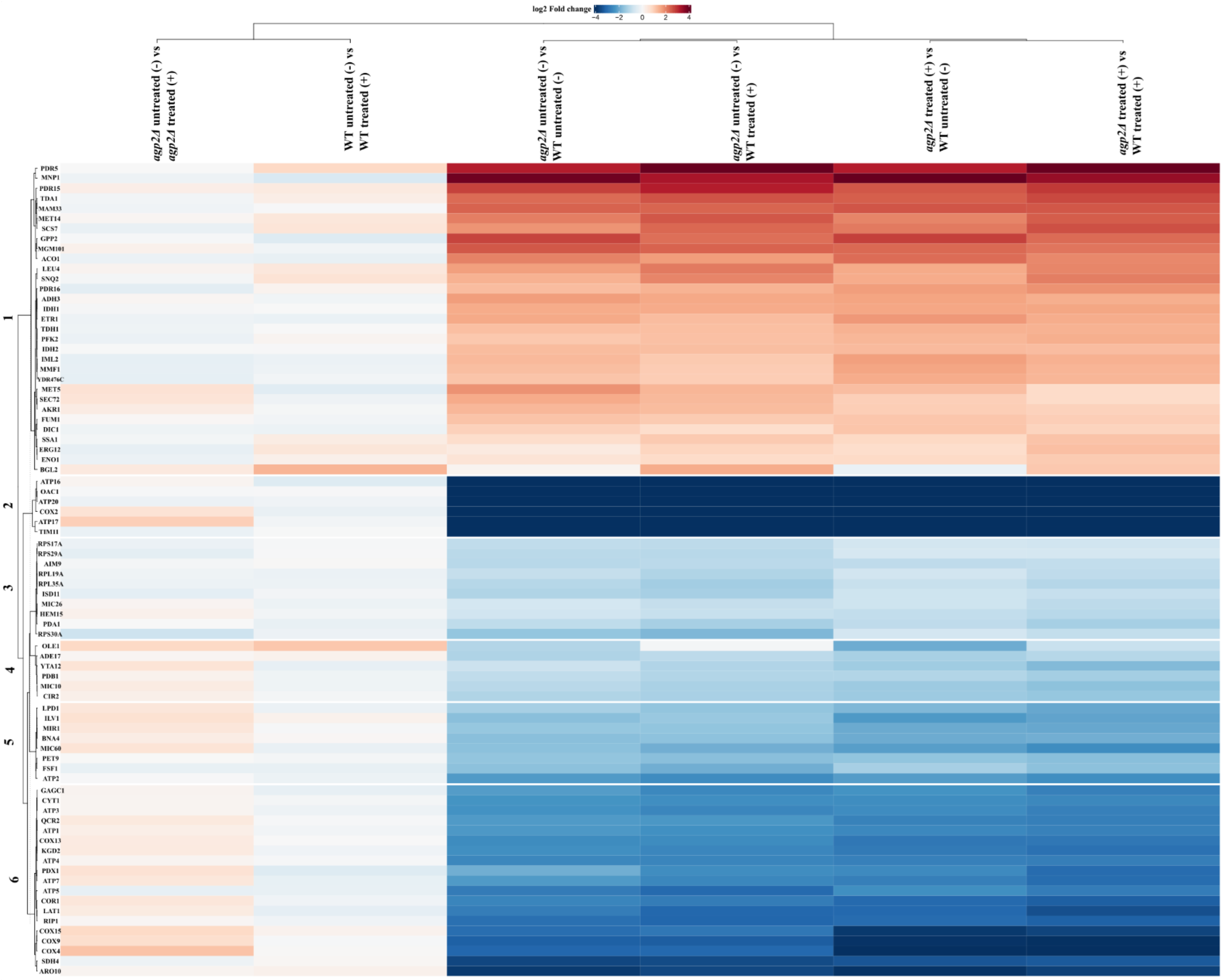

Supplementary Figure S3: Data-centered Heatmap for contrasts. Significant differentially expressed proteins are visually represented as k-means clustered, protein-wise data-centered heatmap for the contrasts. Similar sample contrasts are clustered into column-based clusters while proteins with similar expression patterns are clustered into row-based clusters. Since the parameter $k = 6$ was used, proteins were enriched into 6 repertoires.
