# Supplementary material for: Loss of the yeast transporter Agp2 upregulates the pleiotropic drug-resistant pump Pdr5 and confers resistance to the protein synthesis inhibitor cycloheximide
Source: PLoS One. 2024 May 22;19(5):e0303747. doi: 10.1371/journal.pone.0303747 (PMC11111045; doi:10.1371/journal.pone.0303747)
Supplement: S4 Fig — (PDF) [file pone.0303747.s004.pdf]

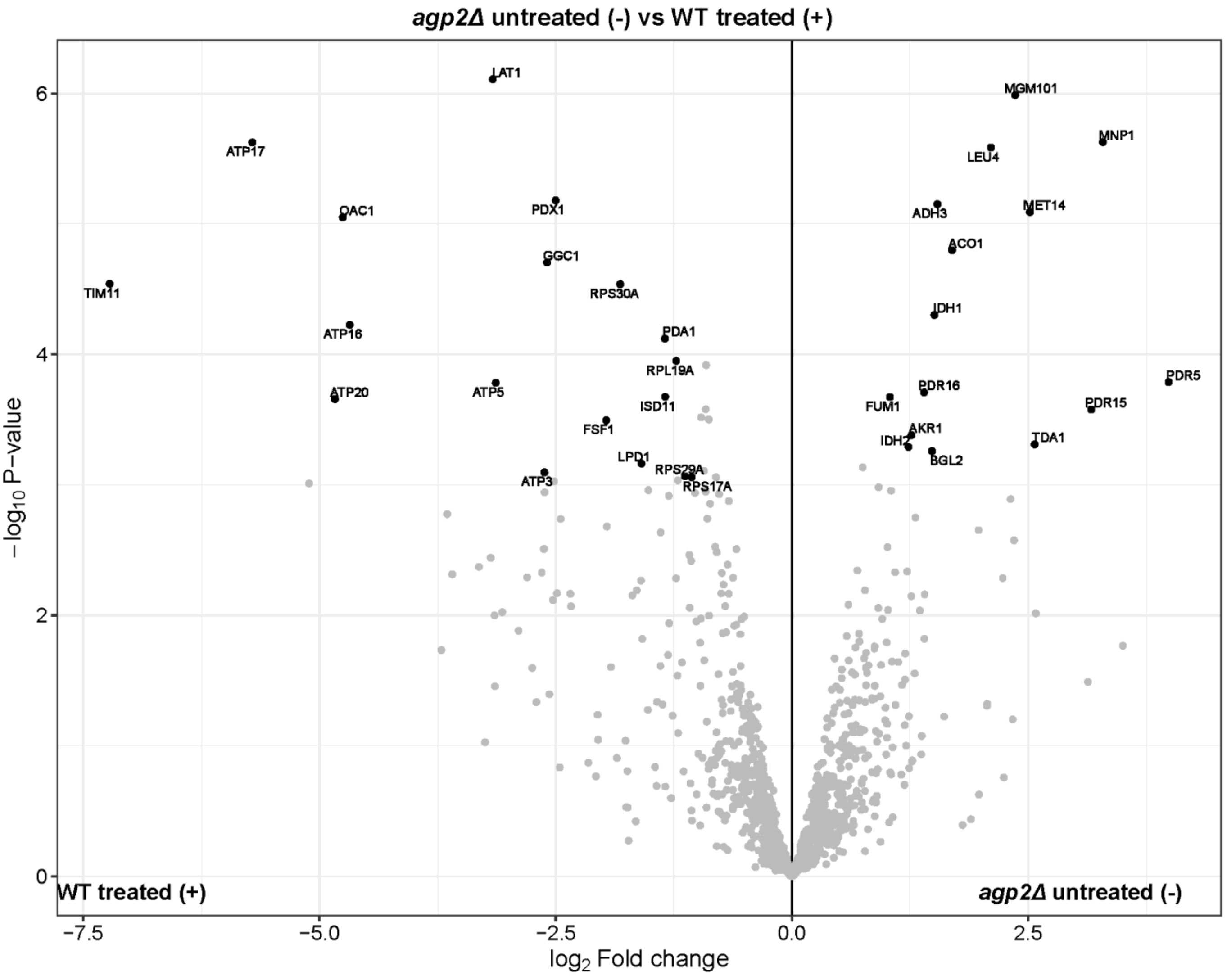

Supplementary Figure S4: Volcano-plot of *agp2Δ* untreated (-) vs. WT treated (+). The gene names of significant differentially expressed proteins with log2 fold change > 0.5 and -log10 P-value > 2 are labelled.
