# Supplementary material for: Loss of the yeast transporter Agp2 upregulates the pleiotropic drug-resistant pump Pdr5 and confers resistance to the protein synthesis inhibitor cycloheximide
Source: PLoS One. 2024 May 22;19(5):e0303747. doi: 10.1371/journal.pone.0303747 (PMC11111045; doi:10.1371/journal.pone.0303747)
Supplement: S8 Fig — (PDF) [file pone.0303747.s008.pdf]

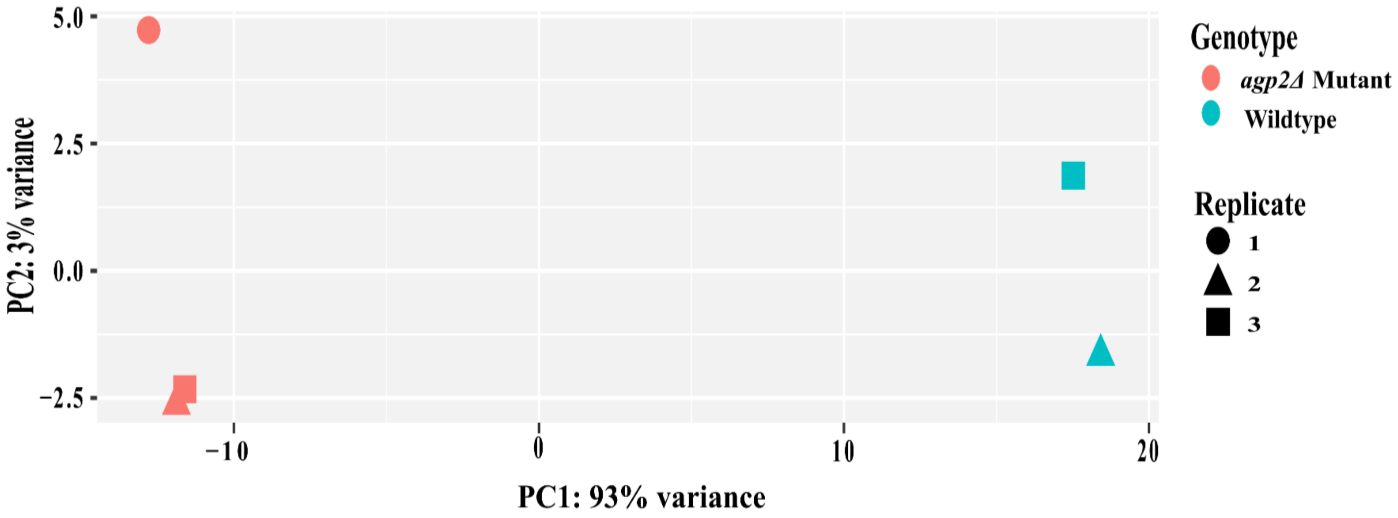

Supplementary Figure S8: Principal components analysis (PCA) plot. The gene counts of 5744 protein-coding genes derived by RNAseq across all the samples were used to generate a PCA plot. Each genotype and replicate are represented with a different colour and shape, respectively, as shown in the legend.
