# Supplementary material for: Loss of the yeast transporter Agp2 upregulates the pleiotropic drug-resistant pump Pdr5 and confers resistance to the protein synthesis inhibitor cycloheximide
Source: PLoS One. 2024 May 22;19(5):e0303747. doi: 10.1371/journal.pone.0303747 (PMC11111045; doi:10.1371/journal.pone.0303747)
Supplement: S9 Fig — (PDF) [file pone.0303747.s009.pdf]

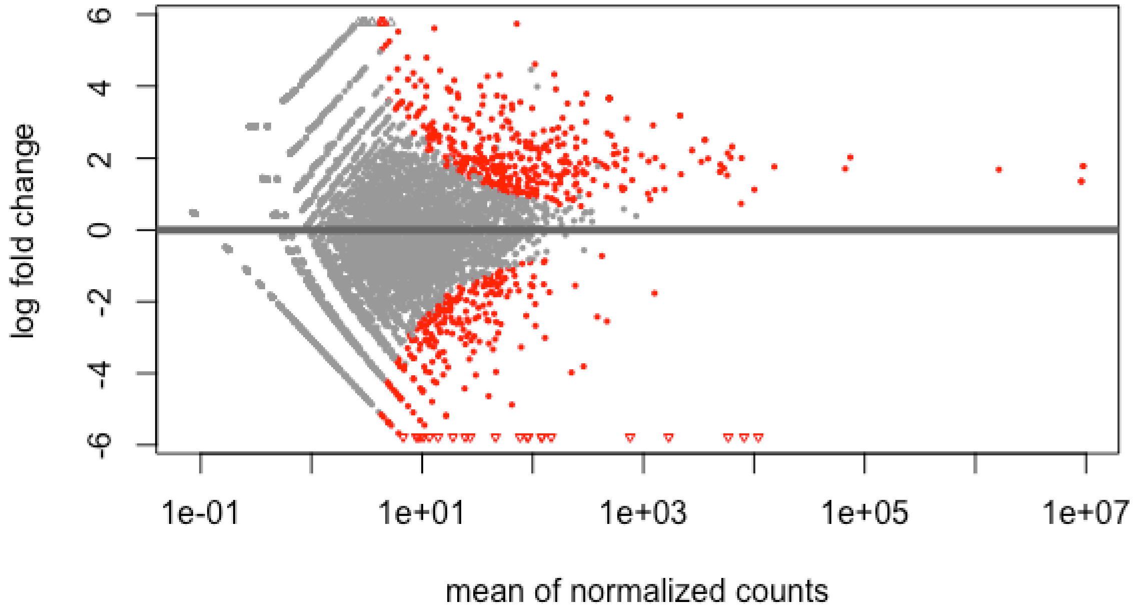

Supplementary Figure S9: MA plot showing the log fold change of 5744 protein coding genes with respect to their mean gene expression between the two strains (derived by DESeq2 differential testing) are represented in the MA plot. The data points highlighted in red are the genes with adjusted *p*-value less than 0.1.
