# Supplementary material for: Loss of the yeast transporter Agp2 upregulates the pleiotropic drug-resistant pump Pdr5 and confers resistance to the protein synthesis inhibitor cycloheximide
Source: PLoS One. 2024 May 22;19(5):e0303747. doi: 10.1371/journal.pone.0303747 (PMC11111045; doi:10.1371/journal.pone.0303747)
Supplement: S10 Fig — (PDF) [file pone.0303747.s010.pdf]

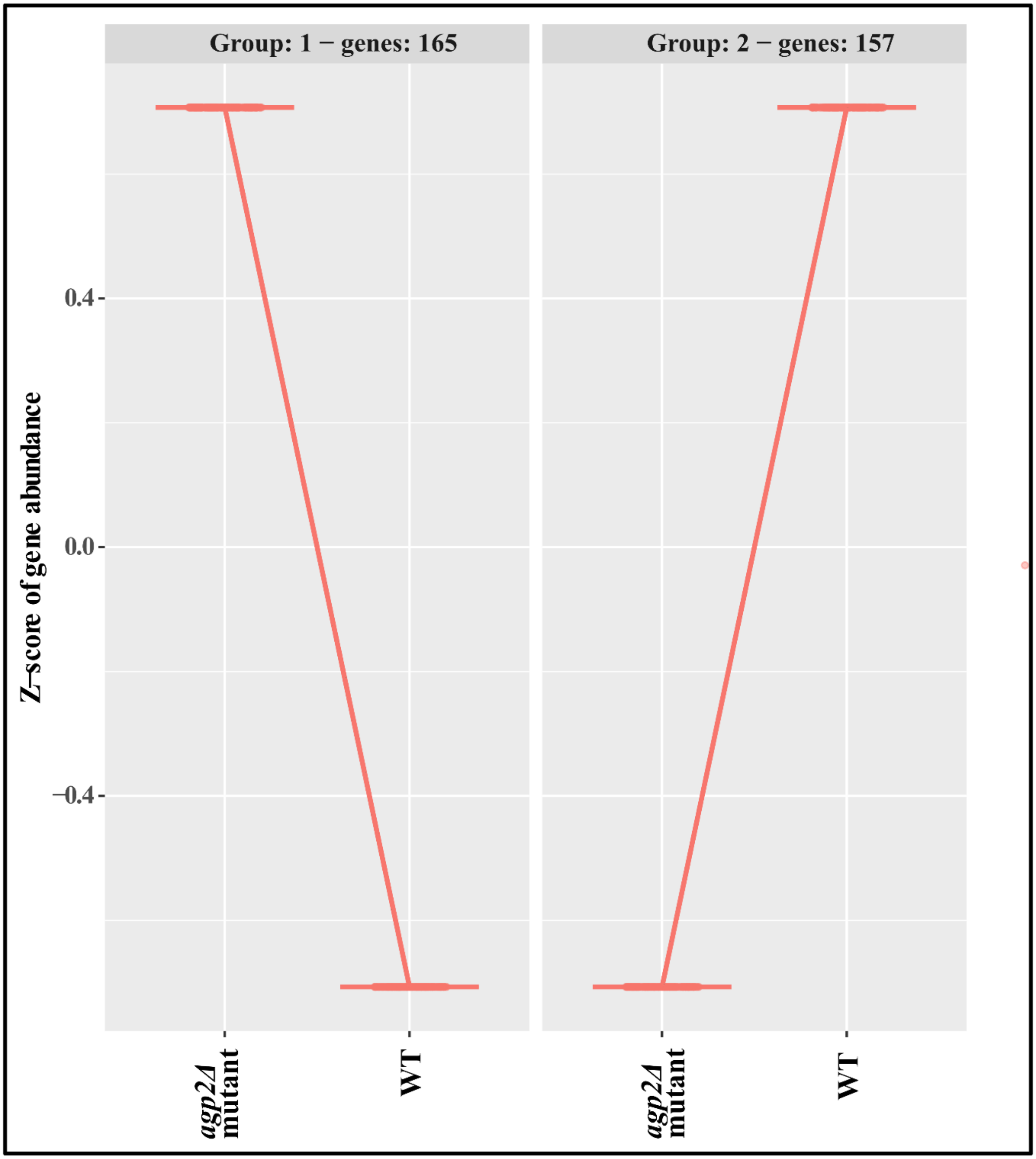

Supplementary Figure S10: The Z-score of gene abundance analysis shows the significant and differentially expressed genes were clustered into 2 groups based on their expression pattern. Group 1: upregulated in *agp2Δ* mutant and Group 2: downregulated in *agp2Δ* mutant.
