# Supplementary material for: Loss of the yeast transporter Agp2 upregulates the pleiotropic drug-resistant pump Pdr5 and confers resistance to the protein synthesis inhibitor cycloheximide
Source: PLoS One. 2024 May 22;19(5):e0303747. doi: 10.1371/journal.pone.0303747 (PMC11111045; doi:10.1371/journal.pone.0303747)
Supplement: S12 Fig — (PDF) [file pone.0303747.s012.pdf]

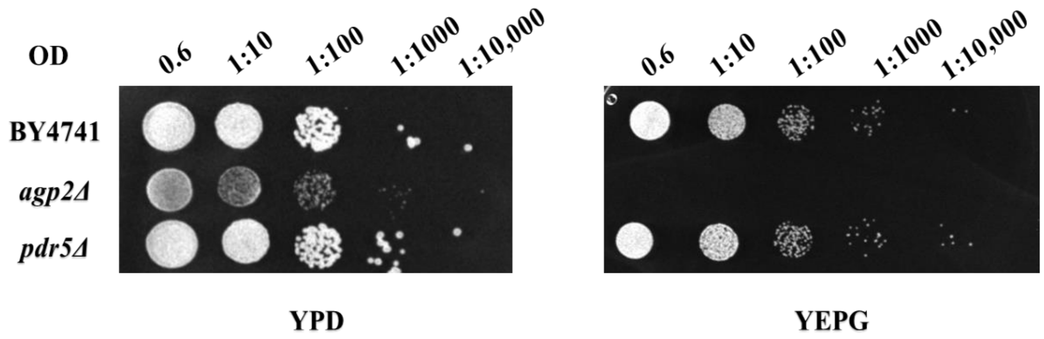

Figure S12: Spot test analysis showing that the *agp2Δ* mutant is unable to grow on solid YEPG media as compared to the WT strain BY4741. Exponentially growing BY4741, *agp2Δ and pdr5*Δ strains were diluted to OD $_{600}$ ~0.6. These cells were serially diluted and spotted onto YPD (normal media) and YEPG (non-fermentable carbon) agar. The plates were incubated at 30 °C and photographed after 48 hrs.
