# Supplementary material for: Loss of the yeast transporter Agp2 upregulates the pleiotropic drug-resistant pump Pdr5 and confers resistance to the protein synthesis inhibitor cycloheximide
Source: PLoS One. 2024 May 22;19(5):e0303747. doi: 10.1371/journal.pone.0303747 (PMC11111045; doi:10.1371/journal.pone.0303747)
Supplement: S13 Fig — (PDF) [file pone.0303747.s013.pdf]

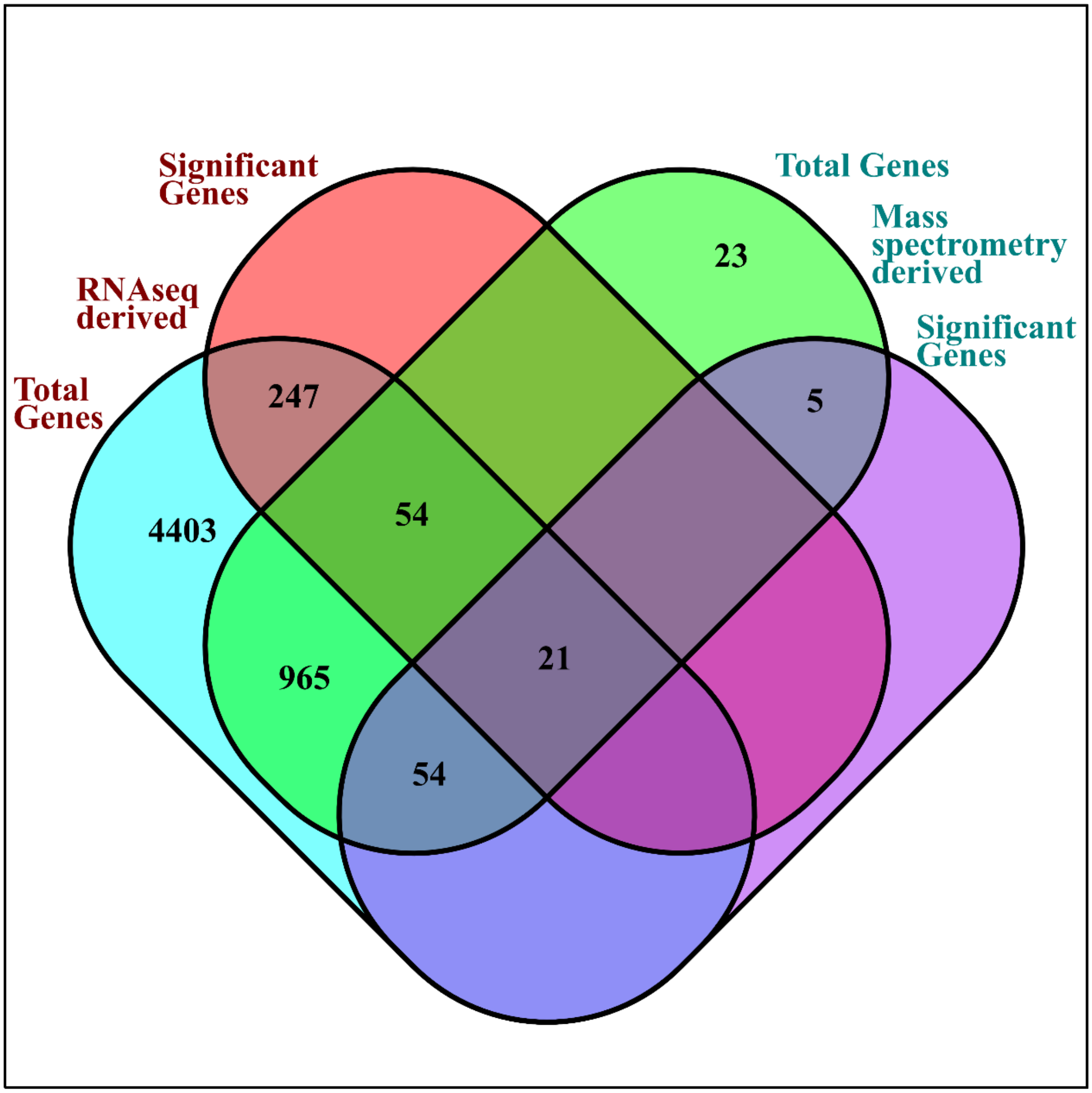

Supplementary Figure S13: The Venn diagram represents the number of identified genes common among the mass-spectrometry and RNAseq-derived dataset. Mass spectrometry uniquely identified 23 proteins while RNA-sequencing uniquely identified 4403 genes.
