# Supplementary material for: Loss of the yeast transporter Agp2 upregulates the pleiotropic drug-resistant pump Pdr5 and confers resistance to the protein synthesis inhibitor cycloheximide
Source: PLoS One. 2024 May 22;19(5):e0303747. doi: 10.1371/journal.pone.0303747 (PMC11111045; doi:10.1371/journal.pone.0303747)
Supplement: S14 Fig — (PDF) [file pone.0303747.s014.pdf]

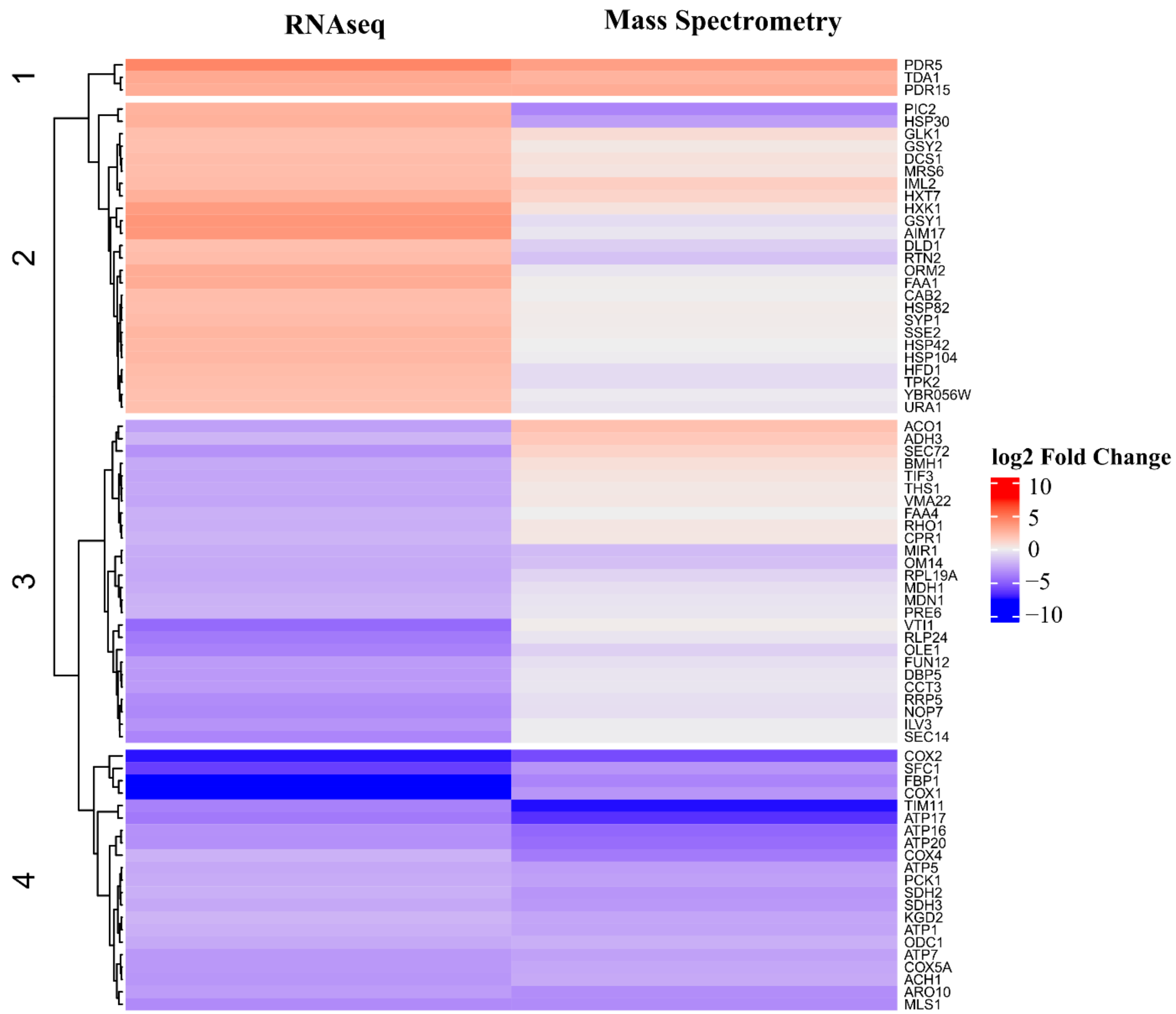

Supplementary Figure S14: Heatmap of log2 Fold Change. Illustration of log2 fold change values of the differentially expressed genes commonly identified from both RNA-sequencing and mass spectrometry data analysis. The log2 fold change values of ‘differentially expressed genes’/ ‘differentially abundant proteins’ was deduced from the contrast data analysis of *agp2Δ* mutant vs. wild type sample dataset derived by ‘RNA-sequencing’/ ‘mass spectrometry’. Genes with similar expression patterns are clustered into row-based clusters. Cluster 1: up-regulated both transcriptionally (high mRNA level detected by RNA-sequencing) and translationally (high protein level detected by mass spectrometry dataset); Cluster 2: up-regulated transcriptionally but moderately down-regulated translationally; Cluster-3: down-regulated transcriptionally but moderately up-regulated translationally; Cluster-4: down-regulated both transcriptionally and translationally.
