# Supplementary material for: Loss of the yeast transporter Agp2 upregulates the pleiotropic drug-resistant pump Pdr5 and confers resistance to the protein synthesis inhibitor cycloheximide
Source: PLoS One. 2024 May 22;19(5):e0303747. doi: 10.1371/journal.pone.0303747 (PMC11111045; doi:10.1371/journal.pone.0303747)
Supplement: S15 Fig — (PDF) [file pone.0303747.s015.pdf]

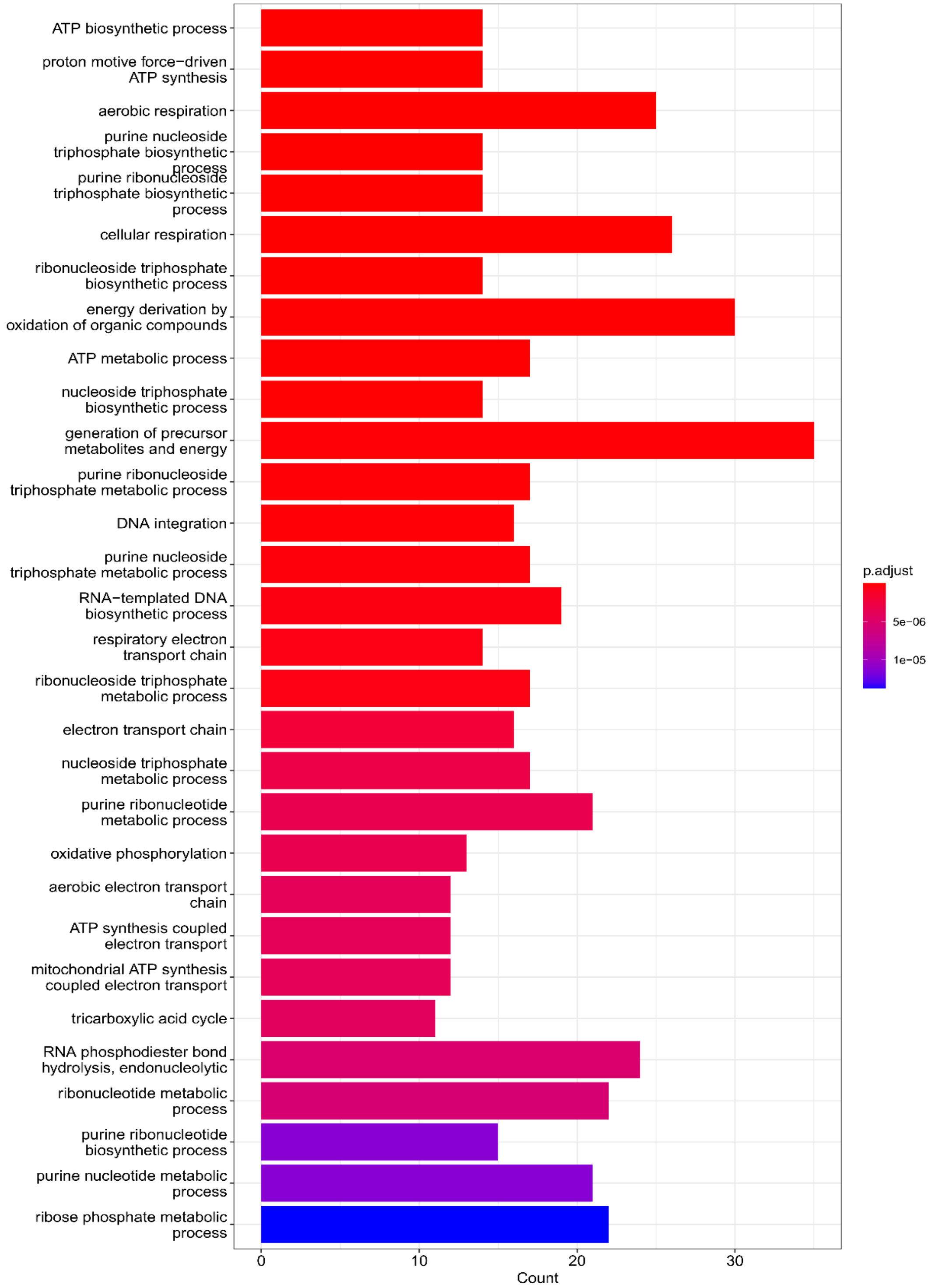

Supplementary Figure S15: Bar plot of enriched GO ontologies for bioprocesses. Top 30 GO ontologies for bioprocesses that were significantly enriched in the pairwise comparisons of *agp2Δ* mutant and wildtype sample groups.
