# Supplementary material for: Loss of the yeast transporter Agp2 upregulates the pleiotropic drug-resistant pump Pdr5 and confers resistance to the protein synthesis inhibitor cycloheximide
Source: PLoS One. 2024 May 22;19(5):e0303747. doi: 10.1371/journal.pone.0303747 (PMC11111045; doi:10.1371/journal.pone.0303747)
Supplement: S4 Table — (DOCX) [file pone.0303747.s019.docx]

**Supplementary Table S4.** Summary of the 57 differentially expressed mitochondrial proteins mapped to the list of upregulated proteins identified by Francesca *et al.*

| Mitochondrial protein | 🡩 Ethanol vs Diauxic | 🡩 Diauxic vs Glucose | 🡩 Ethanol vs Glucose |
| --- | --- | --- | --- |
| **🡩 WT Total** | **3** | **34** | **29** |
| AIM9 |  | 1 |  |
| ATP1 |  | 1 | 1 |
| ATP16 |  | 1 | 1 |
| ATP17 |  | 1 | 1 |
| ATP2 |  | 1 | 1 |
| ATP20 |  | 1 | 1 |
| ATP3 |  | 1 | 1 |
| ATP4 |  | 1 | 1 |
| ATP5 |  | 1 | 1 |
| ATP7 |  | 1 | 1 |
| BNA4 |  |  |  |
| CIR2 |  | 1 | 1 |
| COR1 |  | 1 | 1 |
| COX13 |  | 1 | 1 |
| COX15 |  | 1 |  |
| COX2 |  | 1 | 1 |
| COX4 |  | 1 | 1 |
| COX9 |  | 1 | 1 |
| CYT1 |  | 1 | 1 |
| FSF1 | 1 |  |  |
| GGC1 |  |  |  |
| HEM15 |  |  |  |
| ILV1 | 1 |  |  |
| ISD11 |  |  |  |
| KGD2 |  | 1 | 1 |
| LAT1 |  | 1 | 1 |
| LPD1 |  | 1 | 1 |
| MIC10 |  | 1 | 1 |
| MIC26 |  | 1 | 1 |
| MIC60 |  | 1 | 1 |
| MIR1 |  | 1 | 1 |
| OAC1 | 1 |  |  |
| PDA1 |  | 1 |  |
| PDB1 |  | 1 |  |
| PDX1 |  | 1 | 1 |
| PET9 |  | 1 | 1 |
| QCR2 |  | 1 | 1 |
| RIP1 |  | 1 | 1 |
| SDH4 |  | 1 | 1 |
| TIM11 |  | 1 | 1 |
| YTA12 |  | 1 |  |
| **🡩 agp2Δ Total** | **0** | **10** | **7** |
| ACO1 |  | 1 | 1 |
| ADH3 |  | 1 |  |
| DIC1 |  |  |  |
| ENO1 |  |  |  |
| ETR1 |  | 1 | 1 |
| FUM1 |  | 1 | 1 |
| IDH1 |  | 1 | 1 |
| IDH2 |  | 1 | 1 |
| IML2 |  |  |  |
| LEU4 |  | 1 | 1 |
| MAM33 |  | 1 | 1 |
| MGM101 |  |  |  |
| MMF1 |  | 1 |  |
| MNP1 |  | 1 |  |
| TDH1 |  |  |  |

**Supplementary Table S4: Summarized Table.** The 57 differentially expressed mitochondrial proteins that were mapped onto the list of upregulated proteins identified by Francesca *et al* upon significance testing of pairwise comparisons between the metabolic stages of yeast cells, namely, glucose phase, diauxic shift and ethanol phase. Each column represent the respective pairwise comparison and each row is indexed by the respective gene name of the protein. If a gene name is mapped onto the list of proteins upregulated in a pairwise comparison, it is counted as 1.
