# Supplementary material for: Loss of the yeast transporter Agp2 upregulates the pleiotropic drug-resistant pump Pdr5 and confers resistance to the protein synthesis inhibitor cycloheximide
Source: PLoS One. 2024 May 22;19(5):e0303747. doi: 10.1371/journal.pone.0303747 (PMC11111045; doi:10.1371/journal.pone.0303747)
Supplement: S6 Table — (DOCX) [file pone.0303747.s021.docx]

| Genotype | Sample ID | RNA-Seq reads | BioSample accession |
| --- | --- | --- | --- |
| Wildtype | WT-2 | 45,172,588 | SAMC962608 |
|  | WT-3 | 42,582,618 | SAMC962609 |
| *agp2Δ* mutant | MT-1 | 37,469,822 | SAMC962610 |
|  | MT-2 | 32,445,768 | SAMC962611 |
|  | MT-3 | 30,521,205 | SAMC962612 |

**Supplementary Table S6:** Bulk RNA sequencing statistics
