# Supplementary material for: Loss of the yeast transporter Agp2 upregulates the pleiotropic drug-resistant pump Pdr5 and confers resistance to the protein synthesis inhibitor cycloheximide
Source: PLoS One. 2024 May 22;19(5):e0303747. doi: 10.1371/journal.pone.0303747 (PMC11111045; doi:10.1371/journal.pone.0303747)
Supplement: S1 Graphical abstract — (DOCX) [file pone.0303747.s029.docx]

**Graphical Abstract**


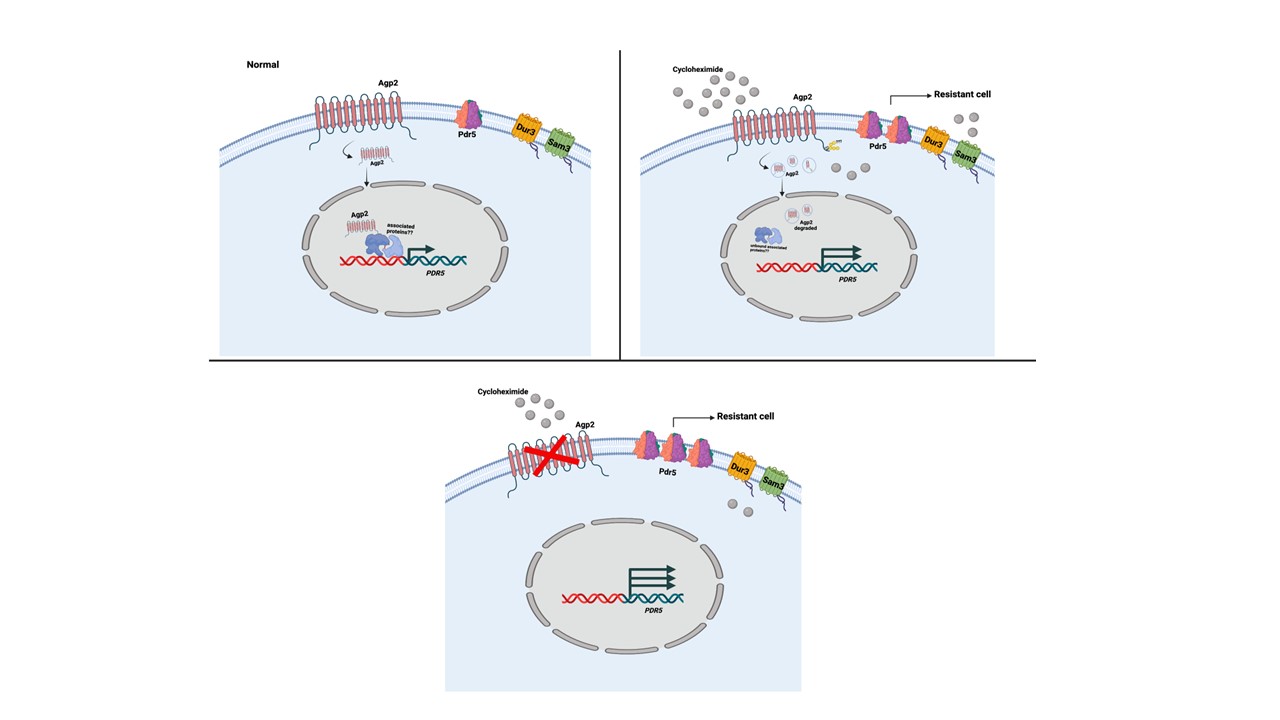


- **Agp2 activates the transport of CHX into the cells**
- **In the absence of Agp2, the drug efflux pump Pdr5 is upregulated**
- **Loss of Agp2 confers hyper-resistance to CHX via Pdr5 upregulation**
