# Supplementary material for: Loss of the yeast transporter Agp2 upregulates the pleiotropic drug-resistant pump Pdr5 and confers resistance to the protein synthesis inhibitor cycloheximide
Source: PLoS One. 2024 May 22;19(5):e0303747. doi: 10.1371/journal.pone.0303747 (PMC11111045; doi:10.1371/journal.pone.0303747)
Supplement: S1 Raw images — (PDF) [file pone.0303747.s030.pdf]

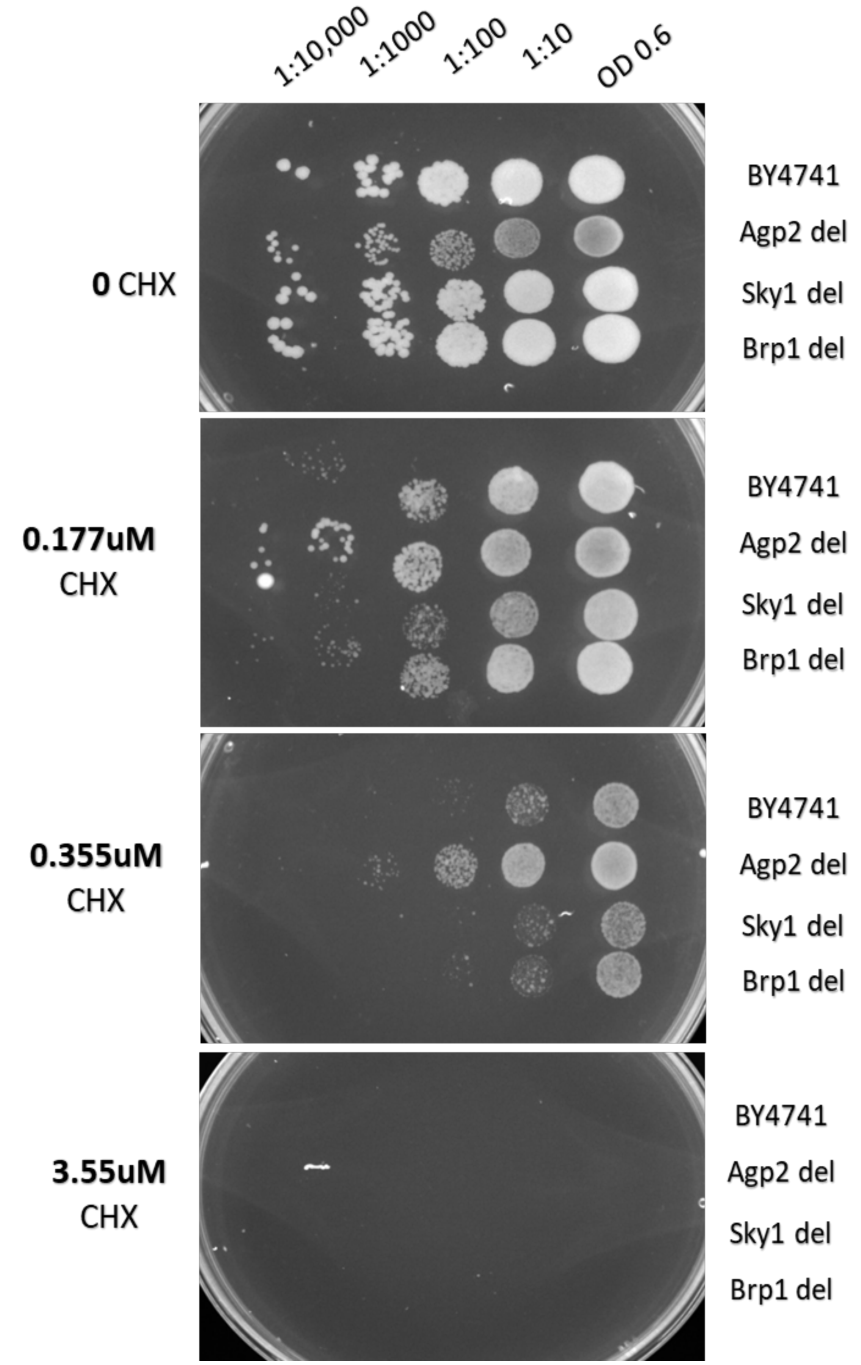

Raw data for Fig. 1A. Expt 1

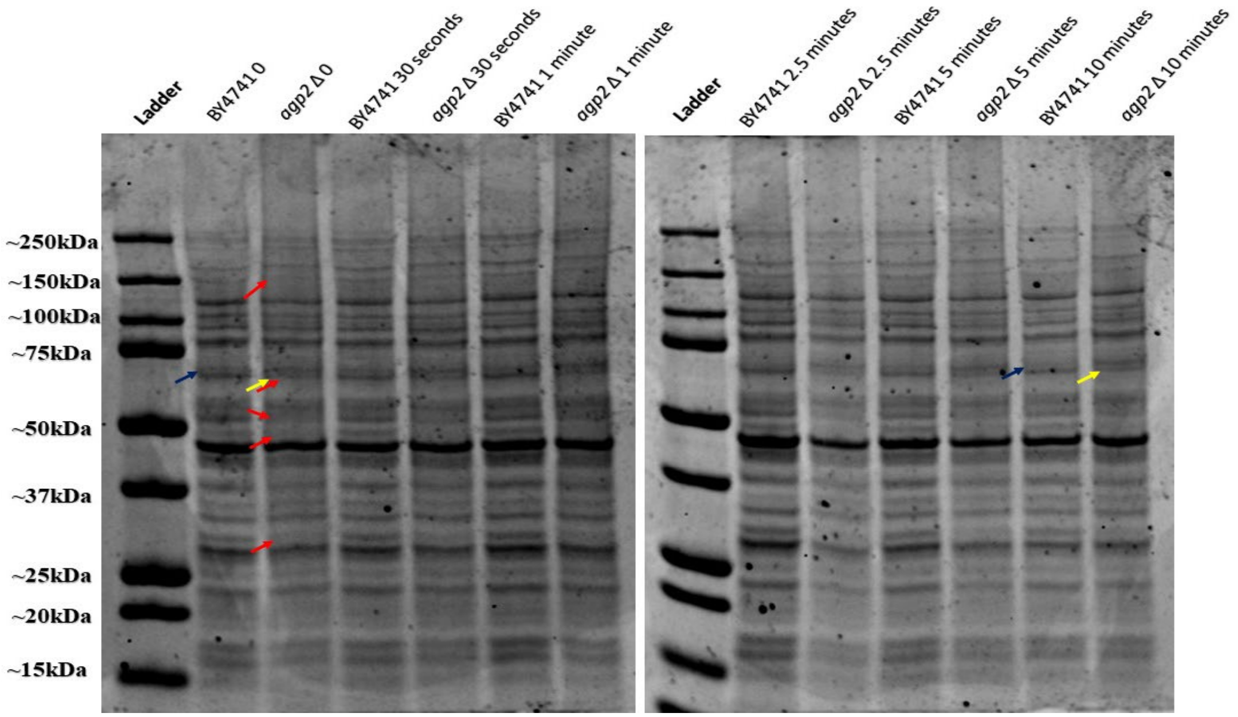
Ladder
BY4741 0
agp2 Δ 0
BY4741 30 seconds
agp2 Δ 30 seconds
BY4741 1 minute
agp2 Δ 1 minute
Ladder
BY4741 2.5 minutes
agp2 Δ 2.5 minutes
BY4741 5 minutes
agp2 Δ 5 minutes
BY4741 10 minutes
agp2 Δ 10 minutes
~250kDa
~150kDa
~100kDa
~75kDa
~50kDa
~37kDa
~25kDa
~20kDa
~15kDa

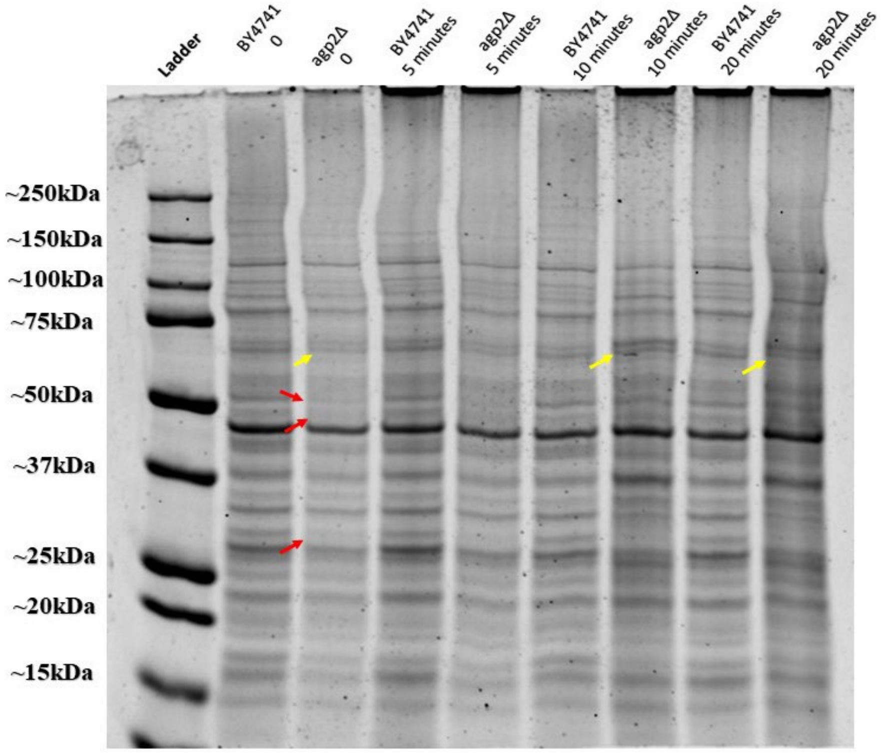
Ladder
BY4741 0
agp2Δ 0
BY4741 5 minutes
agp2Δ 5 minutes
BY4741 10 minutes
agp2Δ 10 minutes
BY4741 20 minutes
agp2Δ 20 minutes
~250kDa
~150kDa
~100kDa
~75kDa
~50kDa
~37kDa
~25kDa
~20kDa
~15kDa

Raw data for Fig. 2-Expt 1, 2., and 4

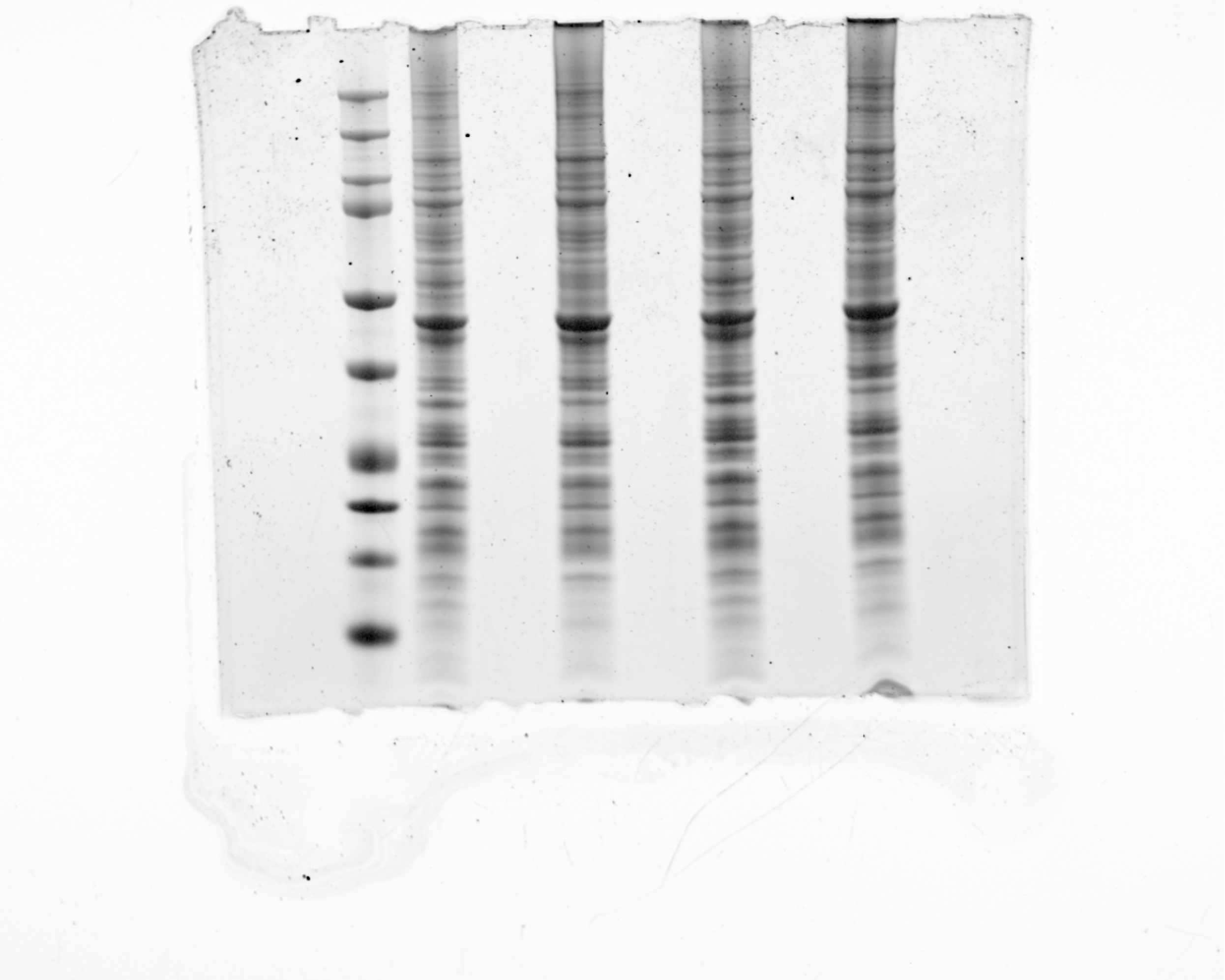

Raw data for Fig. 2-Expt 3

Figure 2: Difference in proteins present on plasma membrane between BY4741 (WT) and *agp2Δ* strains untreated and treated with CHX. Samples were run on 8%SDS-PAGE gel.

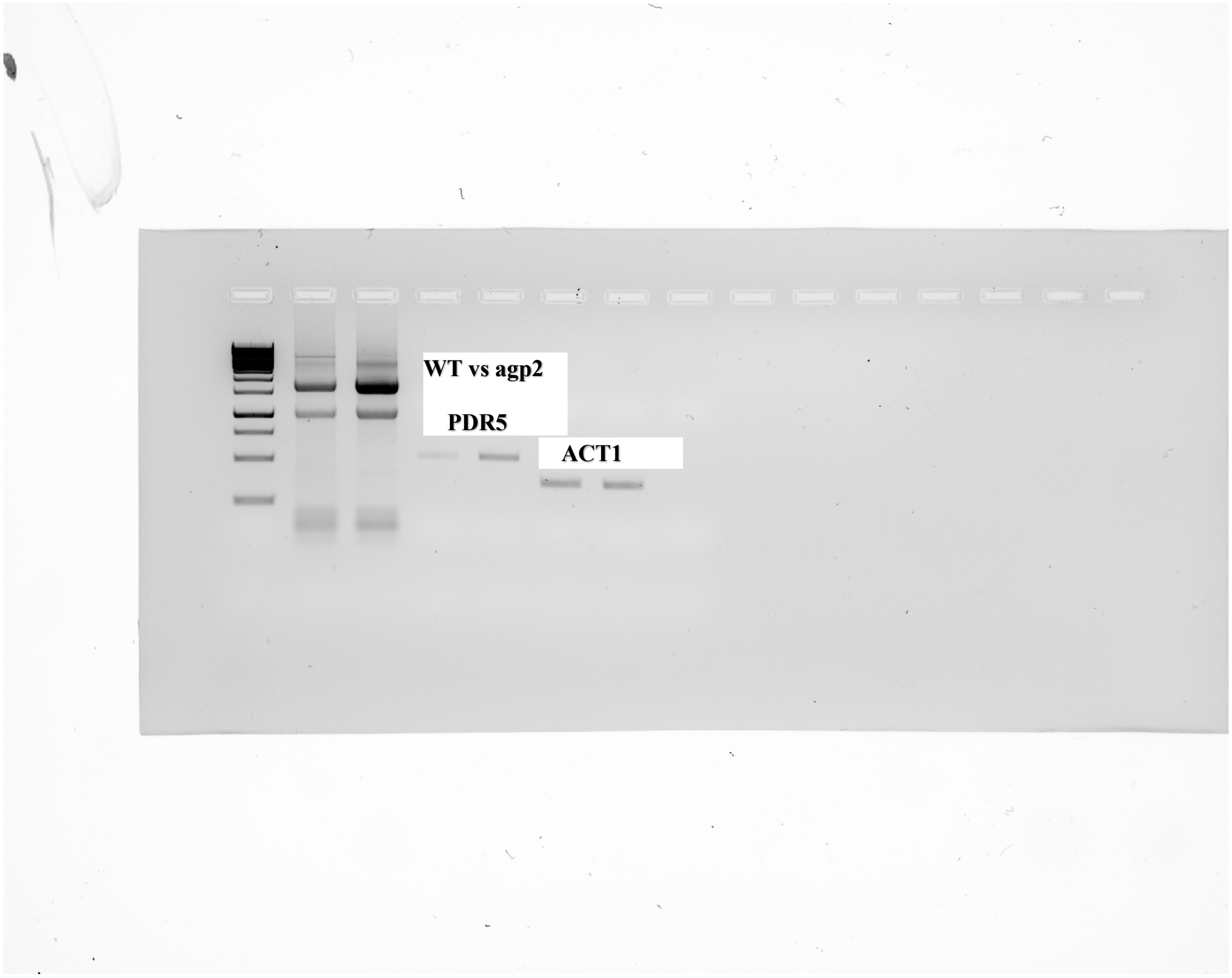

Raw data for Fig. 8A.
