# Supplementary material for: Loss of the yeast transporter Agp2 upregulates the pleiotropic drug-resistant pump Pdr5 and confers resistance to the protein synthesis inhibitor cycloheximide
Source: PLoS One. 2024 May 22;19(5):e0303747. doi: 10.1371/journal.pone.0303747 (PMC11111045; doi:10.1371/journal.pone.0303747)
Supplement: S11 Fig — (PDF) [file pone.0303747.s011.pdf]

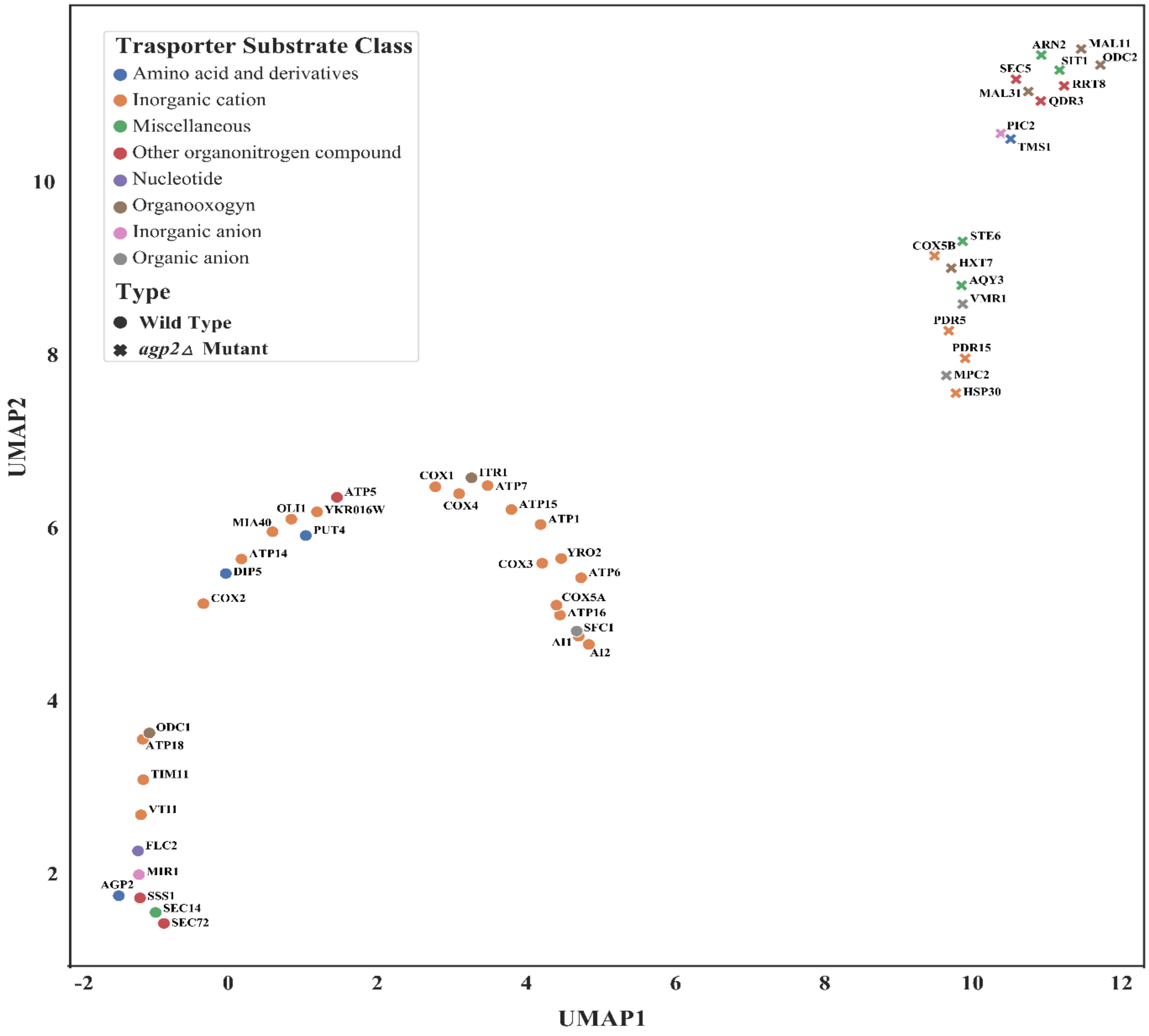

Supplementary Figure S11: UMAP (Uniform Manifold Approximation and Projection) machine learning technique was used for dimension reduction and clustering of significant transporter genes.
