# Supplementary material for: Loss of the yeast transporter Agp2 upregulates the pleiotropic drug-resistant pump Pdr5 and confers resistance to the protein synthesis inhibitor cycloheximide
Source: PLoS One. 2024 May 22;19(5):e0303747. doi: 10.1371/journal.pone.0303747 (PMC11111045; doi:10.1371/journal.pone.0303747)
Supplement: Attachment — Submitted filename: Reponse to Reviewers Apr 1, 2024.docx [file pone.0303747.s031.docx]

Reviewers' comments:
**Reviewer #1:**

The current research work investigates the possible mechanism by which the absence of Agp2 confers resistance to CHX. The work is comprehensively performed using multiple experimental and analytic approaches and is very well-written, introduced and discussed.
The following points need some consideration:

**Comment 1:** The graphical abstract should be improved to include the three sections as shown in Figure 10. It should be more representative and should clearly show that this is a hypothetical model.
Response 1: We have revised the graphical abstract to include the entire Figure 10.

**Comment 2:** Short title should be changed. It is different from the manuscript title and does not reflect the main topic being discussed in the article.
**Response 2:** We have replaced the running title with the following short title: “Role of Agp2 in mediating CHX resistance”.

**Comment 3:** Nomenclature of genes and proteins should be carefully revised and corrected all over the manuscript.

**Response 3:** This has been done.

**Comment 4:** Page 7: Conclusions of the study should not be reported in the introduction section.

**Response 4:** Point well taken. We have modified the statement to read “In addition, we show that Agp2-GFP, and not GFP alone, is associated with the promoter region of the *PDR5* gene using chromatin immunoprecipitation assay implying that Agp2 exerts a regulator role on the *PDR5* gene”.

**Comment 5:** There is detailed description of methodology in results section. Please make sure to remove excess details regarding methods from results and move that to methods section.
**Response 5:** Thank you. We have moved some of the details to the respective Materials and Methods sections.

**Comment 6:** Immunoprecipitation results are mentioned in the discussion but not in the results section.

**Response 6:** It was mentioned under the section entitled “Agp2 associates with the promoter of the PDR5 gene”, which we have now changed to read “Agp2 associates with the promoter of the *PDR5* gene using immunoprecipitation analysis”.

**Comment 7:** Page 33: The conclusion section should be more clear and specific.

**Response 7:** We have modified the conclusion to be more specific.

**Comment 8:** Regarding figure 1B, the symbols on different growth curves and figure legend should be made clearer.

**Response 8:** This is now improved.

**Comment 9:** Page 15 Results section: In interpretation of figure 2, the authors state “suggesting that cells devoid of Agp2 may have defects in regulating protein expression levels.” Could you please clarify this sentence ? What do other arrows “the red, blue, green, and brown arrows” refer to ?
**Response 9:** We have clarified the sentence to read “…devoid of Agp2 may have defects in maintaining the cellular level of specific proteins”. We have alsoreferred to the colour arrows in the text and the legend of Fig. 2.

**Comment 10:** Among the upregulated proteins, why was Pdr5 specifically chosen, was this specifically targeted based on a pre-specified hypothesis to carry out the study? What are other genes of relevance from both mass spectrometry and RNA sequencing results ? Is there any overlap or shared genes ? Is there a priority or importance you find to any of these genes ?

**Response 10:** There are many points here, but briefly we targeted Pdr5 as it was revealed by Mass Spectrometry showing the highest number of unique peptides (28 peptides) and confirmed by RNAseq. More importantly, it was shown to be involved in the efflux of CHX. There are other genes many of which are involved in mitochondrial functions. Our future focus will be to determine the specific factors that allow Agp2 to control mitochondrial function.

**Comment 11:** Page 16: Authors mention “unique peptides and these showed a maximum fold difference of > 4.0” which you include in Supplementary Table S2. Do you find specific importance or physiologic relevance to any of these peptides? I believe this should be included in the discussion section.

**Response 11:** Typically, mass spectrometry results are peptide MS/MS spectra. The observed peptide MS/MS spectra are compared with the theoretical spectra generated from all possible unique peptides in the protein sequence database. The sheer volume of spectra generated by a typical mass spectrometry experiment often leads to protein misidentification. Gilbert et al., (ref - <https://doi.org/10.1002/pmic.200500358>) have noted that >92% of proteins identified by using the criteria of ≥5 unique peptide hits have been replicated across experiments. Therefore, the criteria ≥5 unique peptide hits were used to identify the set of highly true positive proteins and not because of any physiological significance. We have now included a statement and the reference in the revised manuscript.

**Comment 12:** Page 16: Authors also mention that “ four proteins are involved in the multidrug resistance pathway”. Why was Pdr5 specifically included and studied ?

**Response 12:** This point was addressed above in response 10.

**Comment 13:** Page 29 Discussion section: “ we showed by mass spectrometric analysis that the total membrane fractions derived from the agp2Δ mutant contain altered levels of several proteins as compared to the parent strain.” Could you please mention/discuss the most important of these proteins, its possible role, and its possible relation to the current point of study?

**Response 13:** Among the 80 differentially expressed proteins between the WT and the *agp2Δ* mutant, four of these Pdr5 (a multidrug-resistant efflux pump), Pdr15 (a paralog of Pdr5), Pdr16 (a phosphatidylinositol transferase), and Snq2 (a plasma membrane ATP-binding cassette transporter involved in multidrug resistance) are involved in the multidrug resistance pathway and are upregulated in the *agp2Δ*. Pdr5, Pdr15, and Pdr16 are regulated by the multidrug resistance regulator Pdr1 and Pdr3. Pdr5 was the primary focus due to its documented role in multidrug resistance. Of the 80 differentially expressed proteins, 41 are associated with mitochondrial function and are downregulated in the *agp2Δ* mutant. We considered this latter set as the next important group of proteins to be studied, as these are involved in a variety of mitochondrial functions that include transmembrane transport, ion transport, oxidative phosphorylation, ATP synthesis, and cellular respiration. This therefore explains the mitochondrial defect exhibited by the *agp2Δ* mutant, although we do not know the exact mechanism by which Agp2 maintains the expression of the mitochondrial genes. This is now explained in the discussion.

**Comment 14:** Page 28 : Again, authors states that “From these 75 differentially expressed protein-coding genes, 21 were identified as significant in both analyses” . On what bases was Pdr5 efflux pump chosen ?

**Response 14:** This point was addressed above in responses 10 and 13.

**Comment 15:** Page 29: “Unexpectedly, we discovered that the pleiotropic drug efflux pump Pdr5, involved in the extrusion,……….” Why was this unexpected ?

**Response 15:** From our previously published studies, we were expecting Agp2 to regulate an uptake transporter for CHX as it does for polyamines (Aouida et al 2005). However, we found the efflux pump Pdr5 to be upregulated instead. We clarified the “unexpected” in the discussion.

**Comment 16:** Figure 10: I believe that this pathway of action is hypothetical and is a proposed mechanism or a possible interpretation for your findings. As it is not fully experimentally proved, you need to make it clear that this is a hypothesis in your figure title. “ A hypothetical Model”

**Response 16:** The point is well taken. We have now made the changes.

**Comment 17:** Page 33: “and that cells devoid of Agp2 are expected to exhibit a wider range of phenotypes.” What is meant by this sentence ?

**Response 17:** Thank you. We have modified the sentence to read “However, this would imply that Agp2 is associated with positive and negative regulators affecting a number of genes, and as such cells devoid of Agp2 are expected to exhibit resistance not only to CHX, but the responses towards a wider range of drugs.

**Reviewer #2:**

My comments on the manuscript entitled “Loss of the yeast transporter Agp2 upregulates the pleiotropic drug-resistant pump Pdr5 and confers resistance to the protein synthesis inhibitor cycloheximide” are as follows.

**Comment 1:** The study doesn't have clear proof showing that Agp2 is physically connected to the starting area of the PDR5 gene.

**Response 1:** The point is well taken. What we have shown is a clear connection between Agp2 and Pdr5, that is, in the absence of Agp2 the Pdr5 protein and its gene are upregulated as revealed by Mass Spectrometry and RNASeq. In addition, we have shown that Agp2 is associated with the *PDR5* promoter. However, we do not know whether Agp2 directly binds to the *PDR5* promoter or in association with other proteins such as transcription factors. This is the next major phase of the work.

**Comment 2:** The difference in protein levels seen in membrane extracts from agp2Δ mutants might be affected by other things not related to Agp2. Without confirming these effects with other experiments, it's too early to say that the changes are only because Agp2 is missing.

**Response 2:** We are not certain about the comment. However, repeated experiments were always done in comparison with the wild-type under the same conditions, and therefore the only effect would be from the loss of Agp2.

**Comment 3:** The study doesn't consider other ways or backup plans that could also be causing the drug resistance seen in *agp2Δ* mutants. Without looking into these factors, the conclusion about Agp2's role in drug resistance might be too simple.

**Response 3:** We have shown previously that *agp2Δ* mutants are resistant to polyamines and its anticancer analogs, e.g., bleomycin. We have discovered that Agp2 regulates the expression of two high-affinity polyamine uptake transporters Dur3 and Sam3. In the absence of both Dur3 and Sam3 the resulting double mutant *dur3Δ* and *sam3Δ* in not as resistant as the *agp2Δ* mutants, suggesting that indeed other transport mechanism(s) exists to confer drug resistance.

**Comment 4:** The idea that Agp2 breaking down leads to PDR5 going up and causing drug resistance needs more checking with tests that show how things work and with studies that fix the genetics.

**Response 4:** We are not certain about the comment. However, please keep in mind that we cannot create an *agp2Δ pdr5Δ* double mutant (assuming this means “fix the genetics”) as it is not viable, suggesting the importance of the loss of Agp2 to dysregulate Pdr5 levels, although we do not know the specific reason for Pdr5 upregulation-presumably to rid the cells of other toxic metabolites.

**Comment 5:** The in silico evidence provided for the docking of CHX onto Agp2 and its subsequent degradation is based on computational simulations, which may not fully capture the complexities of protein-drug interactions in vivo. Experimental validation through biochemical assays or structural studies is necessary to confirm the binding and degradation kinetics observed.

**Response 5:** We have shown in our recent report by Mohanty et al 2023 the kinetics of Agp2 degradation in response to CHX and that the process requires ubiquitinylation. Structural work on crystallography will require the purification of Agp2 in the absence and presence of CHX. However, this task would require several years of work.

**Comment 6:** While the degradation of Agp2-GFP in response to CHX treatment has been monitored, the exact mechanism of this degradation, including the involvement of ubiquitinylation, needs further experimental verification. Detailed biochemical assays are required to confirm the specific residues involved in ubiquitinylation and the role of Lys63 modification in Agp2 degradation.

**Response 6:** The point is well taken. Please refer to our recently published work by Mohanty et al 2023, which also explored the ubiquitinylation of Agp2. These sites have already been identified.

**Comment 7:** The hypothesis that the degradation of Agp2 in response to CHX exposure reduces the entry of CHX into cells and prevents its toxic effects is plausible but requires direct experimental validation. Additional studies, such as cellular uptake assays with labeled CHX, are needed to confirm the role of Agp2 in CHX transport.

**Response 7**: The point is well taken and we have considered ways to monitor the uptake and efflux of CHX using labeled CHX. However, labeled CHX is not commercially available and one cannot be certain that chemically modified CHX with a fluorescent molecule in a similar manner as we have previously done with the anticancer drug bleomycin will guarantee that the resulting fluorescently labeled form of CHX will retain activity.

**Comment 8:** While RNAseq analysis revealed upregulation of the PDR5 gene in the agp2Δ mutant, the direct repression of PDR5 gene expression by Agp2 and its degradation in response to CHX exposure requires additional experimental validation. Further mechanistic studies, such as chromatin immunoprecipitation assays or reporter gene assays, are necessary to elucidate the regulatory role of Agp2 on PDR5 expression.
**Response 8**: So far, we have shown that Agp2 is associated with the promoter region of the *PDR5* gene using ChIP analysis. We agree we have not fully investigated the mechanism, but this would require extensive work to first find the nuclear protein that is associated with Agp2 that represses *PDR5* gene expression, and then further show that the loss of this repressor would elevate the *PDR5* gene expression independently of Agp2.

**Reviewer #3**:

This group previously reported that Agp2 mutant yeast are resistant to CHX. Agp2 mutation affects the uptake of CHX independently of Dur3 and Sam3. To further investigate the effect of Agp2 mutation, they produced a MS analysis of membrane bound proteins and found that the transporter Pdr5, which is involved in the efflux of CHX, is upregulated in the Agp2 mutant yeast. RNASeq results suggests that the PDR5 gene expression is increased in the Agp2 mutant yeast. They also present results suggesting that Agp2 could bind the promoter region of the PDR5 gene. The Agp2 mutation also affects mitochondrial function.
The manuscript is well written and informative. The results presented are providing new important information on the regulation of different transporters and their connections. The reader would benefit from a better support regarding the knowledge in this field of research.

**Comment 1:** More information regarding the cellular localization of Agp2 and current knowledge on the regulation of Pdr5 protein and PDR5 gene, would improve the manuscript and the global comprehension.

**Response 1**: The point is well taken. One of our major and future undertakings is to unravel the exact mechanism by which Agp2 regulates, specifically the PDR5 gene expression, as well as the 56 mitochondrial genes.

**Comment 2:** Many main figures are presenting general bioinformatic analyses performed on the results. Some data presented in supplementary figures or tables are important. They could be organized and presented in main figures.

**Response 2**: The point is well taken, but we feel that the most important figures are already in the text.

**Comment 3:** Fig 8 and text suggest that the PDR5 transcripts was quantified by RT-PCR but the description in methods suggests that it has been made by qRT-PCR. This should be clarified.

**Response 3**: Thank you. It is RT-PCR for Fig. 8A and qRT-PCR for Fig. 8C and D and we have corrected this in the manuscript.

**Comment 4:** In the model figure, should Agp2 be included in the nuclear membrane, or is it possible that the Agp2 transmembrane domain be disrupted or cleaved?

**Response 4**:. At this stage, we do not know if it is also located on the nuclear membrane or a cleaved fragment that is translocated to the nucleus. It is noteworthy that we are now referring to the model as hypothetical.

**Comment 5:** Discussion regarding the possible reasons or mechanisms potentially involved in the Agp2 nuclear localization and binding to PRD5 promoter would help.
**Response 5**: The point is well taken and we have added to the discussion the following: “However, we have not explored the possibility that the nuclear form of Agp2 is degraded in response to CHX, as we have shown recently for the plasma membrane form [26], in order to promote the expression of the *PDR5* gene”.
