# Supplementary material for: Loss of the yeast transporter Agp2 upregulates the pleiotropic drug-resistant pump Pdr5 and confers resistance to the protein synthesis inhibitor cycloheximide
Source: PLoS One. 2024 May 22;19(5):e0303747. doi: 10.1371/journal.pone.0303747 (PMC11111045; doi:10.1371/journal.pone.0303747)
Supplement: Attachment — Submitted filename: Reponse to Reviewers Apr 24, 2024.docx [file pone.0303747.s032.docx]

Reviewers' comments:
**Reviewer #1:**

PONE-D-24-05515R1

**Comment 1**: In Page 8 in tracked change copy and page 88 in the new PDF revised copy: Conclusions and results of the study has not been removed from the introduction section. Please remove the last paragraph of the introduction that show the results of the study.

**Response 1**: This is now done.

**Comment 2**: Please include the response to comment 10 in the body of the manuscript. **Response 2**: This can be found in the discussion page 29, line 4 from the bottom.

**Comment 3**: Please ensure that the explanation shown in response to comment 13 is included in the discussion section as I cannot find it in tracked change copy.

**Response 3**: This is now included in the discussion see page 30, 2^nd^ para, and top of page 33, 1^st^ para.

**Comment 4**: Again, please make sure that genes and proteins names are correctly formatted all over the manuscript.

**Response 4**: We check every gene and protein.
